# Traumatic Stricture of the Urethra Treated by Resection

**Published:** 1895-08-24

**Authors:** Wm. Horrocks

**Affiliations:** Hon. Surgeon, Bradford Infirmary


### Medical Progress and Hospital Clinics.

[*The Editor will be glad to receive offers of co-operation and contributions from members of the profession. All letters should be addressed to* The Editor, The Lodge, Porchester Square, London, W.]

### TRAUMATIC STRICTURE OF THE URETHRA TREATED BY RESECTION.

By Wm. Horrocks, M.D., F.R.C.S., Hon. Surgeon, Bradford Infirmary.

Wm. F., an insurance agent, aged 52, was admitted to the Bradford Infirmary, suffering from constant dribbling of urine from the urethra. About two and a-half years ago, when mounting a bicycle, he slipped and injured the perinæum. Blood came with the urine for fourteen days, but patient could micturate without the help of instruments. Eventually an abscess formed, and burst externally, urine coming by this opening for many weeks. The fistula closed after some months, the patient passing the urine per urethræm with increasing difficulty. At present the urine issues constantly in drops. When patient attempts to empty the bladder, the urine still only comes in drops, and the effort causes great pain. The urine is alkaline, and contains pus, but no casts. The patient is otherwise in good health. In the perinæum a depressed scar is felt in the right ischio-rectal fossa. The urethra is felt to be thickened in front of the triangular ligament, but the thickening is not considerable, nor is the urethra fixed by cicatricial tissue. On passing a sound into the urethra it comes to a block about five inches from the meatus. Two unsuccessful attempts were made to pass filiform bougies into the bladder. On March 6th, the patient being under ether, a perineal incision was made on the end of a staff passed down the urethra. The urethra was freely divided in its length, and the sides held apart by silk threads. The stricture was then cut through, and a Wheelhouse's director passed into the bladder. The two parts of the urethra had been apparently completely divided at the time of the accident. The anterior end of the first part of the urethra passed directly downwards, while the anterior or penile portion of the urethra travelled forwards. The two parts were united at a sharp angle by a mass of cicatricial tissue, through which there was a narrow canal along which urine passed. This knee or bend in the urethra was removed, and the two parts of the roof of the urethra brought together transversely by interrupted, fine, catgut sutures. The floor of the urethra was repaired by deep and superficial sutures in the length of the urethra. The wound in the urethra was enlarged backwards, and a piece of rubber tube left in to drain the bladder. The front part of the urethra was filled with iodoform emulsion. On the third day the bladder drain was removed, and a large sized Lister's sound (12-15) passed along the whole length of the urethra. Since this time large bougies have been passed at intervals. Urine ceased to come through the perineal wound ten days after the operation, and patient was discharged on March 28th, 1895.

**Remarks.**—The first point of interest is the early history of the case. No instruments were required, although the urethra was almost or quite torn across. It seems noteworthy that a large opening can be made in the urethra in the operation of lithotomy, and the edges of the wounded urethra frequently stretched and bruised, yet no stricture ever follows the operation. The urine in these cases is frequently foul, and must infect the freshly-made wound. On the other hand, in traumatic rupture of the urethra, the parts before the accident are generally healthy, and the urine normal. Yet traumatic rupture, however treated, is almost always followed by troublesome stricture. The reasons for this seem two. In traumatic rupture the urethra is torn across completely, and the periurethral tissues bruised. Secondly, a cavity is formed, connected with the urethra, and distended at intervals by the passage of the urine. From these considerations Mr. Reginald Harrison advises, in all cases of urethral rupture, a perineal incision should be made, whether a catheter can be passed into the bladder or not.

One would rather avoid the passage of a catheter, as a source of infection, and trust to escape of the urine by a large perineal wound. The ideal treatment in this stage would be suture of the ends of the torn urethra and free drainage of the bladder by a posterior opening. This treatment must depend on the amount of bruising about the urethra.

In the case under consideration the objects aimed at were: (1) The restoration of the natural curve of the urethra by removal of the knee or bend caused by the scar; (2) the substitution of a wound healed by first intention for a wound which had healed by granulation. The present condition of the patient is very satisfactory; the largest-sized bougies pass with ease. Even if the divided part of the urethra contracts, the true curve of the urethra is restored, and this greatly assists in passing bougies. The man's condition is now quite natural, and beyond the passage of an occasional bougie, as a precaution, he has no discomfort.

